# LogD7.4 prediction enhanced by transferring knowledge from chromatographic retention time, microscopic pKa and logP

**DOI:** 10.1186/s13321-023-00754-4

**Published:** 2023-09-05

**Authors:** Yitian Wang, Jiacheng Xiong, Fu Xiao, Wei Zhang, Kaiyang Cheng, Jingxin Rao, Buying Niu, Xiaochu Tong, Ning Qu, Runze Zhang, Dingyan Wang, Kaixian Chen, Xutong Li, Mingyue Zheng

**Affiliations:** 1 Drug Discovery and Design Center, State Key Laboratory of Drug Research, Shanghai Institute of Materia Medica, Chinese Academy of Sciences, 555 Zuchongzhi Road, Shanghai, 201203 China; 2 University of Chinese Academy of Sciences, No. 19A Yuquan Road, Beijing, 100049 China; 3 Nanjing University of Chinese Medicine, 138 Xianlin Road, Nanjing, 210023 China; 4 Lingang Laboratory, Shanghai, 200031 China

**Keywords:** logD7.4, Lipid solubility, Graph neural network, Molecular property prediction

## Abstract

Lipophilicity is a fundamental physical property that significantly affects various aspects of drug behavior, including solubility, permeability, metabolism, distribution, protein binding, and toxicity. Accurate prediction of lipophilicity, measured by the logD7.4 value (the distribution coefficient between n-octanol and buffer at physiological pH 7.4), is crucial for successful drug discovery and design. However, the limited availability of data for logD modeling poses a significant challenge to achieving satisfactory generalization capability. To address this challenge, we have developed a novel logD7.4 prediction model called RTlogD, which leverages knowledge from multiple sources. RTlogD combines pre-training on a chromatographic retention time (RT) dataset since the RT is influenced by lipophilicity. Additionally, microscopic pKa values are incorporated as atomic features, providing valuable insights into ionizable sites and ionization capacity. Furthermore, logP is integrated as an auxiliary task within a multitask learning framework. We conducted ablation studies and presented a detailed analysis, showcasing the effectiveness and interpretability of RT, pKa, and logP in the RTlogD model. Notably, our RTlogD model demonstrated superior performance compared to commonly used algorithms and prediction tools. These results underscore the potential of the RTlogD model to improve the accuracy and generalization of logD prediction in drug discovery and design. In summary, the RTlogD model addresses the challenge of limited data availability in logD modeling by leveraging knowledge from RT, microscopic pKa, and logP. Incorporating these factors enhances the predictive capabilities of our model, and it holds promise for real-world applications in drug discovery and design scenarios.

**Graphical Abstract:**

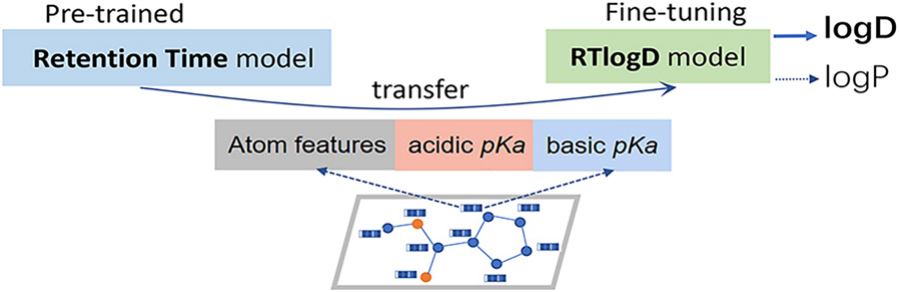

**Supplementary Information:**

The online version contains supplementary material available at 10.1186/s13321-023-00754-4.

## Introduction

Lipophilicity reflects a compound’s ability to dissolve in both octanol and water. In drug-like molecules, lipophilicity affects their physicochemical properties, such as absorption, distribution, metabolism, elimination and toxicology [1, 2]. High lipophilicity has been associated with an increased risk of toxic events, as reported in animal studies conducted by Pfizer [3], while low lipophilicity could limit drug absorption and metabolism [4, 5]. Optimal lipophilicity gives a drug molecule better safety and pharmacokinetic profiles [6]. Therefore, accurately determining the lipophilicity of potential drugs is critical to increasing their chances of success in the development and evaluation processes.

Lipophilicity is generally quantitatively expressed with the n-octanol/water partition coefficient (logP) or the n-octanol/buffer solution distribution coefficient (logD) [1, 7]. LogP describes the differential solubility of a neutral compound with a single form in n-octanol and water. However, 95% of drugs have ionizable groups containing ionization and unionization forms. Thus, logD, which is pH dependent and measures the lipophilicity of an ionizable compound in a mixture of ionic species, is more relevant to drug research. Of particular interest is the logD at the physiological condition pH = 7.4 (logD7.4). According to Bhal’s studies, logD was supposed to be taken into consideration in “Rule of 5” instead of logP [8]. Yang et al. demonstrated that the molecular feature of logD can help distinguish aggregators from nonaggregators in drug discovery [9]. Furthermore, compounds with moderate logD7.4 values exhibit optimal pharmacokinetic and safety profiles, leading to improved therapeutic effectiveness [6]. Overall, logD7.4 plays a crucial role in drug discovery by providing a more comprehensive assessment of a drug’s lipophilicity compared to the commonly used logP value. Accurate prediction of logD7.4 is essential for evaluating drug candidates and optimizing compound properties in the drug discovery process.

Several experimental techniques have been developed to measure logD7.4, including shake-flask, chromatographic and potentiometric approaches. The most commonly used method is the shake-flask method, where n-octanol serves as the octanol phase and buffer acts as the aqueous phase [10]. However, this method is labor-intensive and requires large amounts of synthesized compounds. Chromatographic techniques, particularly high-performance liquid chromatography (HPLC) systems, rely on the distribution behavior between the mobile and stationary phases [11]. Although HPLC method is simple and stable against impurities, it provides an indirect assessment of logD7.4 and is less accurate. Potentiometric titration approaches involve dissolving samples for logD7.4 determination in n-octanol and titrating them with potassium hydroxide or hydrochloride. However, these approaches are limited to compounds with acid–base properties and require high sample purity [12].

Several in silico strategies have been devised to estimate logD due to the complicated experimental determination process. These strategies rely on the quantitative structure–property relationship (QSPR) [13–17]. Artificial intelligence (AI) methods, particularly graph neural networks (GNNs), which use graph representation learning of entire molecules, have been successfully employed in QSPR modeling [18–22]. However, the availability of logD experimental datasets is limited due to proprietary data and the time-consuming shake-flask method, which restricts the generalization capability of the GNNs. To address this, Lapins et al. and Galushka et al. augmented the training datasets with calculated data of nearly 1.6 million predicted logD7.4 values (ACD/logD7.4) from the ChEMBL database [15, 16]. Although this method uses a large amount of data, Fu et al. pointed out that utilizing predicted values can magnify the discrepancy between the predicted and the actual values, leading to suboptimal model performance for new molecules [17].

Pharmaceutical companies have harnessed their proprietary models to predict logD values. In comparison to academic endeavors, these models exhibit superior performance owing to the utilization of their extensive and confidential datasets. Bayer generates thousands of new data points annually [23], AstraZeneca has an expansive in-house database containing experimental drug metabolism and pharmacokinetics values [24], and Merck & Co. is significantly investing in leveraging institutional knowledge to guide their experimental endeavors [25]. Notably, AstraZeneca’s AZlogD74 model is trained on a dataset of over 160,000 molecules, which they continuously update with new measurements [24].

Previous academic studies have incorporated logP and pKa to estimate logD [26, 27], considering the inherent limitations in data quantity and quality for logD predictions. The acid dissociation constant, pKa, represents an equilibrium constant defined as the negative logarithm of the ratio of protonated and deprotonated components in a solvent. Unlike logP, which disregards the molecule's ionization form, pKa provides information about a compound’s ionization state and capacity, which logD takes into account. Therefore, it is important to acknowledge the correlation between logD, logP, and pKa. One theoretical approach assumes that logD can be calculated from logP and pKa [27, 28]. However, this calculation assumes that only the neutral species are distributed in the organic phase, disregarding the fact that octanol can dissolve a significant amount of water, allowing the ionic species to partition into octanol through water. This presence of both charged and uncharged species in the organic phase can lead to a significant error. To address the scarcity of data, data-driven methods such as transfer learning and multitask learning can uncover underlying the contributions of pKa and logP to logD, enabling a more comprehensive and reliable utilization of the data [29, 30]. Wu et al. employed transfer learning using experimental pKa and logP data [26], while Aliagas et al. utilized macroscopic pKa values of molecules predicted by the commercial software Moka as a descriptor of logD [31]. Lukashina et al. and Wieder et al. employed multitask learning to simultaneously learn logD and logP tasks, resulting in improved prediction performance compared to learning the logD task alone [32, 33].

In addition to logP and pKa, chromatographic retention time has also shown a strong correlation with the logD task. Parinet et al. used calculated logD and logP as descriptors to predict retention time [34], highlighting the link between molecular chromatographic behavior and logD. Chromatographic techniques offer rapid high throughput analysis, producing a substantial amount of chromatographic retention time data that surpasses the available logP and pKa data. Win et al. incorporated liquid chromatography retention time as a descriptor to improve the accuracy of logD prediction [35]. However, their dataset only included 2070 molecules, which underutilizes the majority of almost 80,000 molecules in the chromatography retention time dataset [36]. To the best of our knowledge, no previous research that has employed chromatographic retention time as a source task in transfer learning for logD prediction. Including retention time (RT) through transfer learning will expand the molecule dataset, encompassing more compounds and making valuable contributions to the logD task.

In this study, we have established RTlogD, a framework designed to predict the molecule’s logD by integrating relevant information, such as RT, logP and pKa. First, we used RT prediction as a source task by constructing a pre-trained model trained on a dataset of nearly 80,000 molecules. Fine-tuning this RT model enhances the generalization capability of logD prediction because it has been exposed to a large number of molecules. Second, we incorporated logP as an additional task in parallel with logD prediction, creating a multitask model for lipophilicity prediction. The domain information contained in logP task serves as an inductive bias that improves the learning efficiency and prediction accuracy of the logD model. Lastly, we integrated the predicted acidic and basic microscopic pKa values as atomic features. The microscopic pKa of ionizable atoms can offer more specific ionization information, enabling enhanced lipophilicity prediction for different molecular ionization forms. To validate our method, we curated a time-split dataset consisting of molecules reported within the past 2 years and compared the performance of the RTlogD model with widely used tools such as ADMETlab2.0 [14], PCFE [37], ALOGPS [38], FP-ADMET [13] and the commercial software Instant Jchem [39].

## Methods

### Data sets

#### DB29-data

The DB29-data consists of experimental logD values gathered from ChEMBLdb29 [40]. This dataset serves as modeling data due to its comprehensive coverage, facilitating the construction of a logD model with optimal performance. To ensure data quality, it exclusively includes experimental logD values obtained from the shake-flask method, chromatographic techniques, and potentiometric titration approaches. The following pretreatment steps were taken: (1) Records with pH values outside the range of 7.2–7.6 were removed. (2) Records with solvents other than octanol were eliminated. (3) All data was manually verified, and errors were corrected. We identified two types of errors: those resulting from partition coefficient not logarithmically transformed, and transcription errors where the values recorded in ChEMBLdb29 do not align with those in primary literature sources. Rectifying the first type of error is relatively straightforward, as these values can manifest as significantly large and hence are discernible. To address the second type of error, we have endeavored to rectify these discrepancies by cross-referencing the logD records in ChEMBLdb29 with logD values predicted by Instant Jchem. Whenever a record exhibited notable deviations from the predicted logD values, we manually corrected it based on its literature sources. (4) For the same molecule with multiple experimental values that did not significantly vary, the arithmetic mean of these values was adopted as the experimental value for that molecule. Otherwise, the molecule was excluded. (5) Chemical structures were standardized by removing all salts from molecules, computing the normalized tautomer of the molecule, neutralizing charged molecules, and standardizing SMILES strings using the RDKit package [41]. After these pretreatments, a logD7.4 modeling data set with 19,128 compounds was obtained for training models.

#### T-data

To build an external test dataset that has not been used in model training during the comparison with existing logD prediction tools, we also processed the ChEMBLdb32 to create a time-split external test dataset following the same protocol described above. This yielded 2753 newly added logD7.4 data, which were compiled as T-data.

#### Lipo dataset

Additionally, the Lipo dataset was used to conduct a comparative analysis between several GNN-based logD models and RTlogD. Lipo dataset is from MoleculeNet deposited by AstraZeneca and includes 4200 compounds [42], which is widely recognized as a benchmark for logD prediction models. Here, the Lipo dataset was randomly split into the training, validation, and test sets with a ratio of 8:1:1.

#### RT dataset

We gathered the METLIN small molecule retention time (SMRT) data sets as auxiliary data sets to improve logD prediction performance. The SMRT data sets contain chromatographic retention time data for 80,038 small molecules using high-performance liquid chromatography‒mass spectrometry, with values ranging from 0.3 to 1471.7 s. The remaining 79,957 molecules were used to construct chromatographic retention time models after removing molecules with no retention time [36].

#### LogP dataset

A total of 13,553 logP values were collected from PhysProp [43], 2534 logP values from NCI Open Database Compounds [44], 773 values from OChem [45] and 707 logP values from DiverseDataset [46]. After normalization and deduplication, the resulting logP datasets contain 13,688 molecules. Table 1 summarizes the different types of data sets used in this work.

**Table 1:** Different type of data sets used in this work

Type	Dataset	Size
LogD	DB29-data	19,128
	T-data	2753
	Lipo	4200
LogP	PhysProp	13,688
	NCI open	
	OChem	
	DiverseDataset	
Retention time (RT)	SMRT	79,957

The RT dataset, logP dataset, DB29-data, T-data and the Lipo dataset can be found in our GitHub repository.

### Baseline models

In this study, we employed four machine learning algorithms as baseline models for logD prediction: random forest [47] (RF), support vector machine [48] (SVM), artificial neural network [49] (ANN) and extreme gradient boost [50] (XGBoost) (Additional file 1: Fig S1**)**. XGBoost was implemented using the XGBoost package, while SVM, RF and ANN were implemented using the Scikit-learn package [51]. To encode the molecular structures as input to the models, we used Extended connectivity fingerprints (ECFPs) with a diameter 4 and a fingerprint length of 2048 bits. Additionally, we implemented several GNN-based methods to compare our model, including MolMapNet, MGA, StructGNN, KEMPNN, CoMPT, ALipSol and ALipSol + [26].

### Attention-based graph neural network

**Fig. 1:**
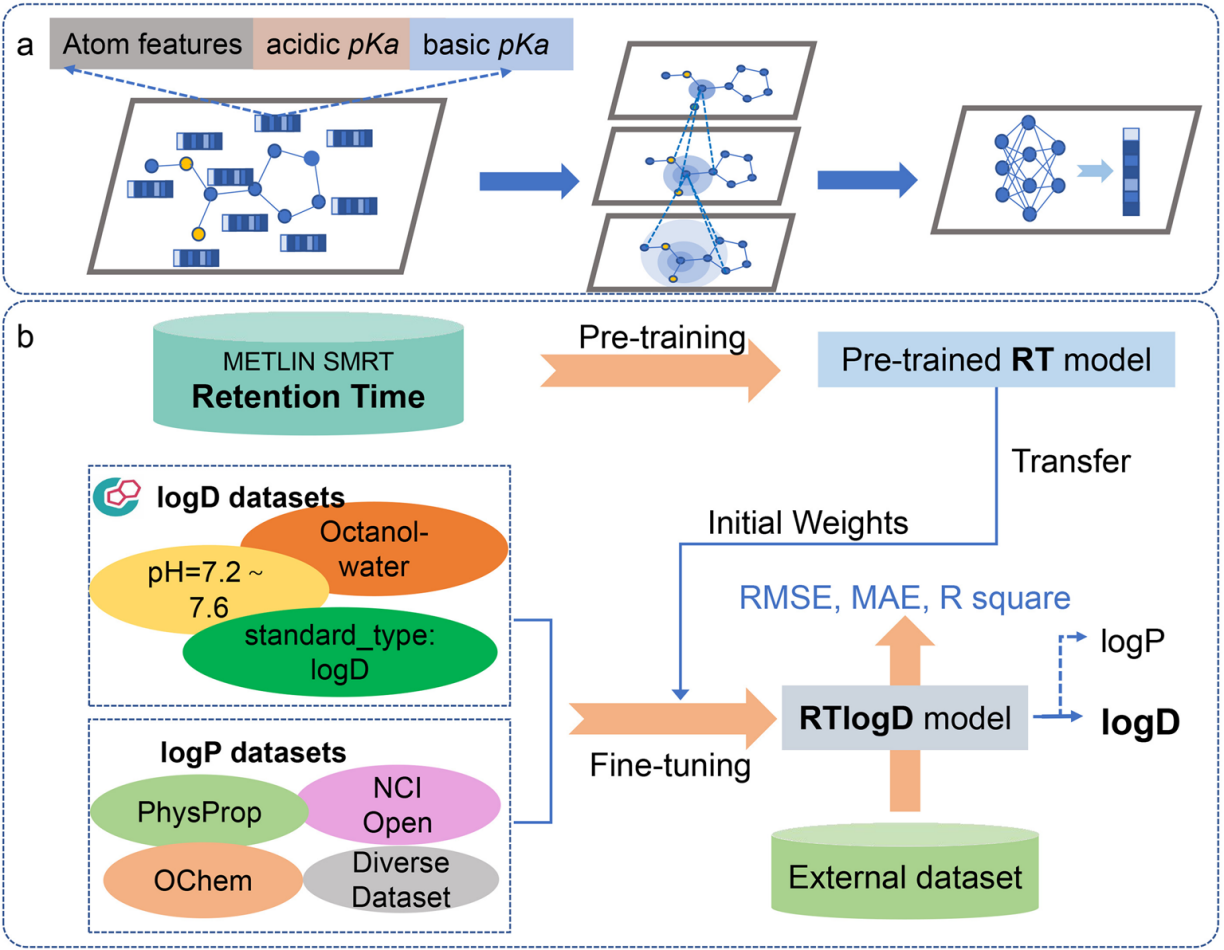
The architecture of the RTlogD model. **a** The graph neural network used in RTlogD. **b** Transfer learning of RT and the multitask learning of logP and logD module

The foundational structure of RTlogD is derived from our previously developed graph attention model called Attentive FP [18], which was implemented using Deep Graph Library (DGL) [52]. This method employs a graph attention mechanism into the graph neural network (GNN) to concentrate on the most relevant parts of the inputs to attain a more favorable prediction. Initially, we utilized the DGL package and RDKit toolkit to convert the molecule's SMILES string into an undirected graph, incorporating nine types of atom features including microscopic pKa values (see “Introducing pKa features”) and four types of bond features (Additional file 1: Table S1). Subsequently, the molecular graph was passed through three graph neural layers to facilitate message passing and update node representations. The readout operation computed graph representations from node features, followed by a multilayer perceptron (MLP) with two fully connected layers to predict graph labels based on the obtained graph features (Fig. 1a).

### Pre-training model of RT

To initialize the network parameters of the logD model, we adopted a pre-training strategy. We initially trained an RT model using the aforementioned attention-based graph neural network and the SMRT dataset. During training, the SmoothL1Loss was used as the loss function. Optimization was performed using Adam with weight decay. Grid searching optimization was applied for hyperparameter tuning, determining the best hyperparameter set for each model based on the validation set. The search ranges and optimal values of these hyperparameters are detailed in Additional file 1: Table S2. To enhance regularization and mitigate neuron co-adaptation, a dropout layer was integrated during training, randomly setting elements in the pooling output vector to zero with a probability of *p* = 0.2. Additionally, batch normalization was applied to expedite and stabilize the training process. The evaluation dataset’s performance was computed after each epoch. Lastly, the weights of the best-performing model were employed as the initial parameters for the subsequent fine-tuning model.

### Multitask learning for logD and logP

We conducted fine-tuning on the pre-trained RT model within a parallel multitask learning architecture [55], aiming to predict logP and logD values simultaneously. In contrast to the single-task attention-based graph neural network employed in the RT model, the multilayer perceptron generates two outputs: one for logD and the other for logP. The hyperparameters remained consistent with those of the pre-trained RT model, except for the learning rate, which was reduced by a factor of 10 to preserve the RT information acquired during pre-training. We combined the compounds from the DB29-data and logP dataset, subsequently allocating them into training, validation, and test sets at an 8:1:1 ratio based on their molecular scaffolds. Early stopping was implemented based on the averaged squared Pearson correlation coefficient for logP and logD tasks on their respective internal validation sets. During training set calculations, for each molecule, we computed the SmoothL1Loss using the available value for either the logD or logP, omitting the unknown value. In cases where both logD and logP values were available, we computed the mean loss for these two tasks.

### Introducing pKa features

We modified GNN model Attentive FP to incorporate molecular pKa features, based on our previously developed multi-instance learning framework Graph-pKa [18, 53]. Specifically, we concatenated the predicted acidic microscopic pKa and basic microscopic pKa as new features at the atomic level in our Attentive FP model. This expanded the initial 74-dimensional atomic feature calculated by RDKit to 76 dimensions. The acidic microscopic pKa is only assigned to non-carbon atoms connected to at least one hydrogen atom, with lower values indicating stronger acidic ionization ability. The basic microscopic pKa is assigned to nitrogen atoms without a positive formal charge, with higher values indicating a stronger basic ionization ability. Both acidic and basic microscopic pKa values were normalized to a range of zero to one. Graph-pKa was also used to predict macro-pKa values of the molecules used for the calculation in CALlogD.

### Evaluation metrics

This study introduced three metrics to assess the model’s performance: the mean absolute error (MAE), root-mean-squared error (RMSE) and R-squared coefficient of determination (R^2^). We also introduced Spearman’s correlation coefficient $({r}_{s}$) to measure the monotonicity of the relationship between two datasets.

1 $$MAE = \frac{1}{n}\sum\limits_{i = 1}^{n} {{\text{|}}y_{i} - \hat{y}_{i} {\text{|}}}$$

2 $$RMSE = \sqrt {\frac{1}{n}\,\sum\limits_{i = 1}^{n} {\left( {y_{i} - \hat{y}_{i} } \right)^{2} } }$$

3 $$R^{2} \, = \,1 - \frac{{\sum\nolimits_{i = 1}^{n} {\left( {y_{i} - \hat{y}_{i} } \right)^{2} } }}{{\sum\nolimits_{i = 1}^{n} {\left( {y_{i} - \overline{y}} \right)^{2} } }}$$

4 $$r_{s} = \frac{{{\text{cov}} \left( {{\text{R}}\left( X \right),{\text{R}}\left( Y \right)} \right)}}{{\sigma_{{{\text{R}}\left( X \right)}} \sigma_{{{\text{R}}\left( Y \right)}} }}$$

In Eqs. (1) through (3), ${y}_{i}$ and ${\widehat{y}}_{i}$ are the measured and predicted values for the molecule *i,* respectively, and $\overline{y }$ is the mean of all molecules in the datasets. In Eq. (4), $\mathrm{cov}\left(\mathrm{R}\left(X\right),\mathrm{R}\left(Y\right)\right)$ is the covariance of the rank variables, ${\sigma }_{\mathrm{R}\left(X\right)}$ and ${\sigma }_{\mathrm{R}\left(Y\right)}$ are the standard deviations of the rank variables.

## Results and discussion

### The implementation of RTlogD

The implementation strategies of RTlogD are presented in Fig. 1, which comprises two main parts. The first part, shown in Fig. 1a, illustrates an attention-based graph neural network that incorporates molecular pKa features, which is the backbone structure of RTlogD (see “Method”).

The second part, shown in Fig. 1b, depicts the overall workflow of RTlogD, including pre-training on RT and multitask learning for logD and logP. To initialize the logD model’s network parameters, we first pre-trained an RT model (see “Method”). We evaluated our pre-trained RT model against the current state-of-the-art (SOTA) model, GNN-RT [54]. We observed that our model achieved satisfactory performance, comparable to the best model to date (Additional file 1: Table S7). Then, we fine-tuned the pre-trained RT model within a parallel multitask learning architecture, aiming to predict logP and logD values simultaneously (see “Method”).

### Performance evaluation

To evaluate the predictive performance of RTlogD, we compared it with the theoretical method and GNN-based logD models. The GNN-based logD models and RTlogD were trained on the Lipo training/validation sets and evaluated on the Lipo test set (see “Method”). The theoretical method, known as CALlogD, is derived from the predicted logP and pKa values using Eq. 5 to estimate logD7.4 [28].

5 $$\log D_{{\left( {PH} \right)}} = \log P - \log \left( {1 + 10^{{\left. {pH - pK_{a} } \right)\delta_{i} }} } \right)$$

where δ_i_ = {1, − 1} for acids and bases, respectively. In this method, the prediction of logD requires logP and pKa as known parameters for input. Here, logP is predicted by the auxiliary task of RTlogD and pKa is predicted by Graph-pKa.

The performance of the RTlogD model and the baseline models are presented in Table 2. Among all the methods, the CALlogD method displayed the poorest performance, which can be attributed to the inherent approximation error in the formula itself, as well as the error accumulation arising from the prediction of logP and pKa. Our RTlogD model outperformed all other baseline models, achieving an R^2^ of 0.835, MAE of 0.341, and RMSE of 0.505. The second-best model, ALipSol + , leverages prior knowledge of pKa, logS, and logP for model construction, indicating the effectiveness of transfer learning. Compared to ALipSol + , as well as other models, our model utilized more prior knowledge of RT and more molecular structural information through pre-training, resulting in superior performance.

**Table 2:** Different model performances on the Lipo Data Set

Model	R^2^	Lipo (N = 4200)	
		MAE	RMSE
CALlogD	− 0.251	0.869	1.356
MolMapNet	0.685 ± 0.036	0.501 ± 0.025	0.682 ± 0.040
MGA	0.768 ± 0.030	0.423 ± 0.022	0.585 ± 0.044
StructGNN	0.791 ± 0.020	0.374 ± 0.019	0.556 ± 0.035
KEMPNN	0.767 ± 0.027	0.410 ± 0.018	0.589 ± 0.040
CoMPT	0.767 ± 0.032	0.417 ± 0.020	0.588 ± 0.046
ALipSol	0.813 ± 0.028	0.362 ± 0.019	0.526 ± 0.048
ALipSol +	0.820 ± 0.025	0.349 ± 0.021	0.516 ± 0.045
RTlogD	**0.835 ± 0.026**	**0.341 ± 0.018**	**0.505 ± 0.044**

Values in bold represent the superior performance among the various methods

### Comparison with logD prediction tools

**Fig. 2:**
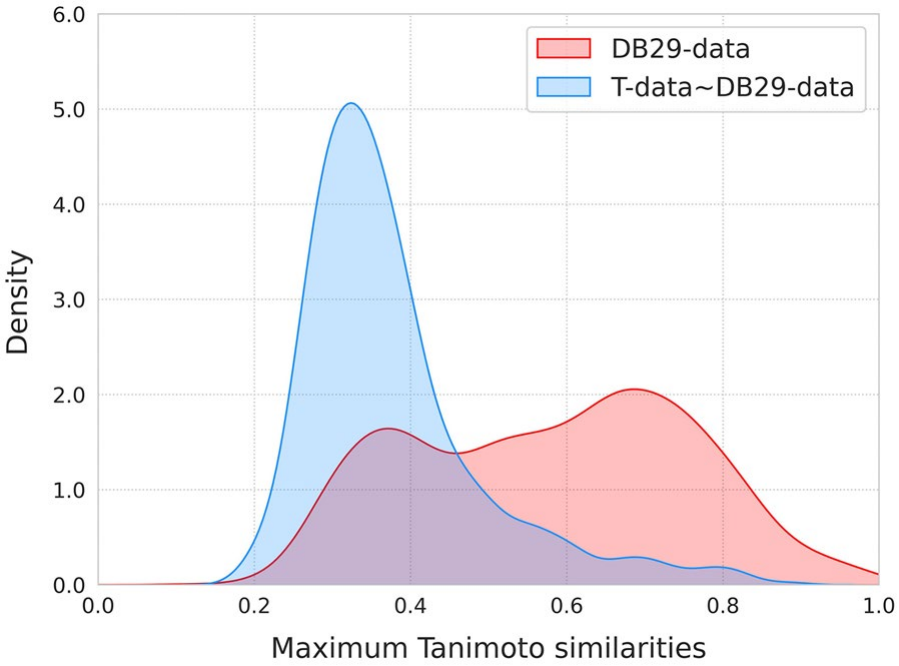
Comparison of the maximum Tanimoto similarities distribution within DB29-Data (red), and between T-Data and DB29-Data (blue) using ECFP4

To further investigate the performance of RTlogD in the logD prediction task, we conducted a more stringent time-split evaluation, to ensure that the newly collected test data have not been exposed to the model training. We trained RTlogD using 19,128 logD samples in the ChEMBLdb29 database (DB29-data) and tested it on the newly disclosed samples in ChEMBLdb32, which consists of 2753 recently measured logD values (T-data) (see “Method”). On the one hand, T-data provides a relatively fair benchmark to compare RTlogD with existing logD prediction tools, such as ADMETlab2.0, PCFE, ALOGPS, FP-ADMET and Instant Jchem. On the other hand, in terms of the discrepancy observed between T-data and DB29-data within the chemical space, T-data is valuable to assess the generalization capability of the RTlogD model. To assess the structural dissimilarity between the T-data and DB29-data, we employed the molecular fingerprint ECFP4 [56] to calculate both the max internal similarities within DB29-data and the max similarity of each molecule in T-data relative to DB29-data. Figure 2 illustrates that most molecules in T-data show structural dissimilarity compared to DB29-data, as evidenced by low max similarities ranging from 0.2 to 0.4. This enabled us to perform an independent evaluation and comparison of various predictive tools based on T-data.

We conducted a comparison between our proposed RTlogD model and five commonly used tools: Instant Jchem, ADMETlab2.0, PCFE, FP-ADMET and ALOGPS. The results presented in Table 3 clearly demonstrate the significant advantages of RTlogD over other tools. RTlogD exhibited a higher R^2^ value and a lower RMSE and MAE value, indicating its superior performance. The PCFE model ranked as the second-best model, which utilized 1.71 million computational logD values for pre-training before fine-tuned with experimental logD7.4 data. In contrast, our model achieved superior results using only approximately 80,000 chromatographic data for pre-training. Despite having a smaller pre-training dataset compared to PCFE, our model’s performance suggests that incorporating auxiliary information, such as logD, logP, and pKa, through reasonable training strategies effectively contributes to its superior performance. Therefore, we further investigated the individual contributions of different modules RT, logP, and pKa, to the final prediction performance.

**Table 3:** Comparison with existing prediction tools on T-data

Task	Model	Performance metrics		
		R^2^	MAE	RMSE
logD7.4	Instant Jchem	− 1.094	1.121	1.991
	ADMETlab2.0	0.309	0.856	1.144
	PCFE	0.495	0.701	0.978
	FP-ADMET	0.067	1.033	1.329
	ALOGPS	− 0.049	1.054	1.409
	RTlogD	**0.550**	**0.694**	**0.923**

Values in bold represent the superior performance among the various methods

### Ablation experiments

We first conducted ablation studies to evaluate the impact of auxiliary information on the logD prediction performance of the RTlogD model. Specifically, we examined the model’s performance on T-data when it was not pre-trained on the RT dataset, did not incorporate microscopic pKa as atom features, or did not include the logP multitask. Table 4 presents the comparison of the complete RTlogD model with variations that exclude certain components. Additionally, Additional file 1: Table S6 presents the performance of various logD prediction model variations, including incorporating all auxiliary information as multitask, using logP as the pre-training task while employing RT as multitask, and substituting macroscopic pKa for microscopic pKa.

**Table 4:** Comparison with ablated models on T-data

Task	Model	RMSE	MAE	R^2^
logD7.4	RTlogD	**0.923**	**0.694**	**0.550**
	w/o RT*	0.996	0.731	0.476
	w/o microscopic pKa	0.997	0.734	0.475
	w/o RT and microscopic pKa	1.046	0.758	0.422
	w/o logP	0.952	0.717	0.521

^*^w/o denotes “without”

Table 4 shows that the “w/o RT” model, “w/o microscopic pKa” model, “w/o RT and microscopic pKa” model, and “w/o logP” model had a decrease in logD prediction performance. This emphasizes the importance of incorporating auxiliary information from RT, microscopic pKa features, and logP to enhance the overall performance of the RTlogD model. Notably, the RTlogD model, which combines pre-training on the RT dataset, incorporation of microscopic pKa as atomic features, and inclusion of the logP multitask, outperformed other strategies (Additional file 1: Table S6) and achieved the highest level of performance.

The effectiveness of QSPR models usually relies heavily on the similarity between the predicted molecules and those in the training set. This relationship is evident in Additional file 1: Fig S2, where the model’s prediction accuracy improves as the molecules in the T-data become more similar to those in the training set. Pre-training on RT data, which is closely related to logD, can address the challenge of data scarcity for logD modeling and enhance the generalization ability for predicting novel molecules.

To verify this, we conducted an experiment to gradually expand the chemical space of the training dataset with and without RT pre-training, respectively. Various numbers of molecules were sampled from the DB29-data as training data to train a series of models with expanding chemical space. The T-data was then used to evaluate the prediction performance of these models.

Specifically, to simulate out-of-domain prediction tasks commonly encountered in real-world applications, we adopted a prioritized sampling approach for selecting molecules from the DB29-Data that are less similar to the T-Data. For each molecule in the T-Data, we calculated its maximum Tanimoto similarity with molecules in the DB29-Data using ECFP4 fingerprints. Subsequently, we sorted the molecules in the DB29-Data in ascending order based on their similarity scores. The top N molecules were then sampled to construct a series of models. The initial model was built using the top 1000 molecules with the lowest similarity scores, while the subsequent models were constructed using the top 2000, 4000, 6000, 8000, 10,000, 1,2000, and 1,4000 molecules by incrementing the dataset size by 2000 compounds at each step.

**Fig. 3:**
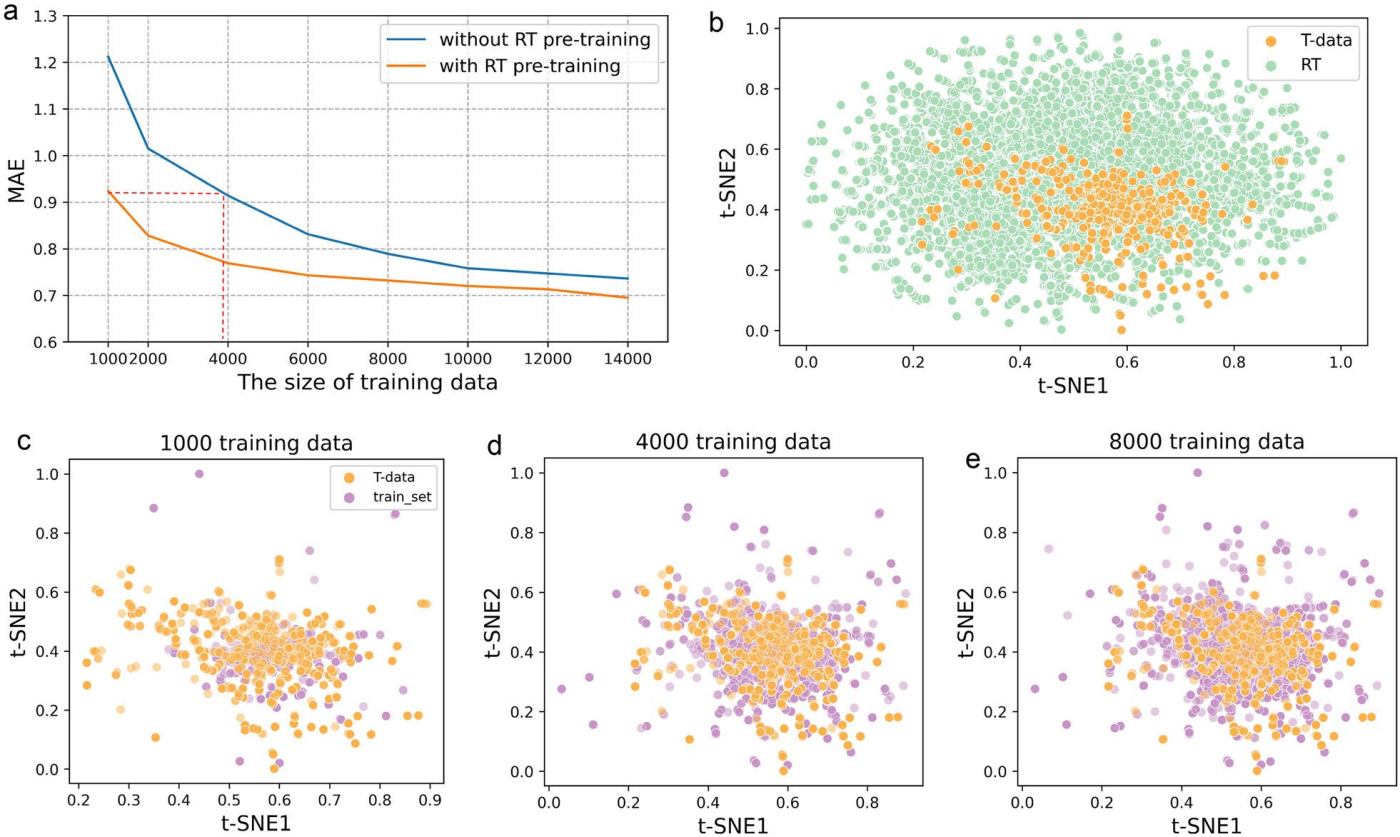
Effect of training data size on the prediction performance of T-data. **a** Model performance variation with and without RT pre-training. **b** t-SNE distribution of T-data and RT by ECFP4. **c** t-SNE distribution of T-data and 1000 training data sampled from DB29-data by ECFP4. **d** t-SNE distribution of T-data and 4000 training data. **e** t-SNE distribution of T-data and 8000 training data

The variation in prediction performance with respect to the size of the training data is depicted in Fig. 3a. Overall, the prediction performance improved as the training data size increased, regardless of whether RT pre-training was used or not. This improvement can be attributed to the data-hungry nature of GNNs, which require more data to fit the model and prevent overfitting. However, the performance improvement achieved by adding thousands of molecules in models without RT pre-training can be attained by simply incorporating an RT pre-training operation. As shown in Fig. 3a, enhancing the logD training data size from 1000 to 4000 leads to improved performance, with a decreased MAE from 1.212 to 0.914 for models without RT. When utilizing the RT pre-trained strategy, good performance can be achieved (MAE = 0.924) with only 1000 logD training data (the red dashed line in Fig. 3a). These results suggest that incorporating RT as pre-training may reduce the number of instances required for model training. Furthermore, with a smaller training data size, the performance gap between the models with and without RT pre-training is wider, indicating that the introduction of RT pre-training has a more pronounced impact when dealing with low data volumes and has achieved a notable generalization capability for predicting novel molecules.

In addition, we investigated the reasons behind Fig. 3a and proposed that the RT data enables it to leverage relevant knowledge. To analyze the chemical space of RT and logD dataset, we employed t-distributed stochastic neighbor embedding (t-SNE) [57] based on molecular fingerprints ECFP4. When training with only 1000 molecules, the training set in the DB29-data covers only a fraction of the T-data (Fig. 3c). When training with 4000 molecules, the coverage of the T-data by the training set is increased (Fig. 3d). However, adding more data does not improve the coverage further, as the chemical space of the training set remains relatively constant (Fig. 3e). Meanwhile, the chemical space of RT directly encompasses most of the T-data, except for a small portion representing peptides (scatters at bottom right-hand corner in Fig. 3b). The incorporation of RT exposes the model to a wider range of molecules and improves its inductive bias. These visualization results are consistent with the performance statistics presented in Fig. 3a. Although the performance gaps between the two models diminish as the logD training data size increases, the model with RT pre-training consistently outperforms in terms of generalization capability.

**Fig. 4:**
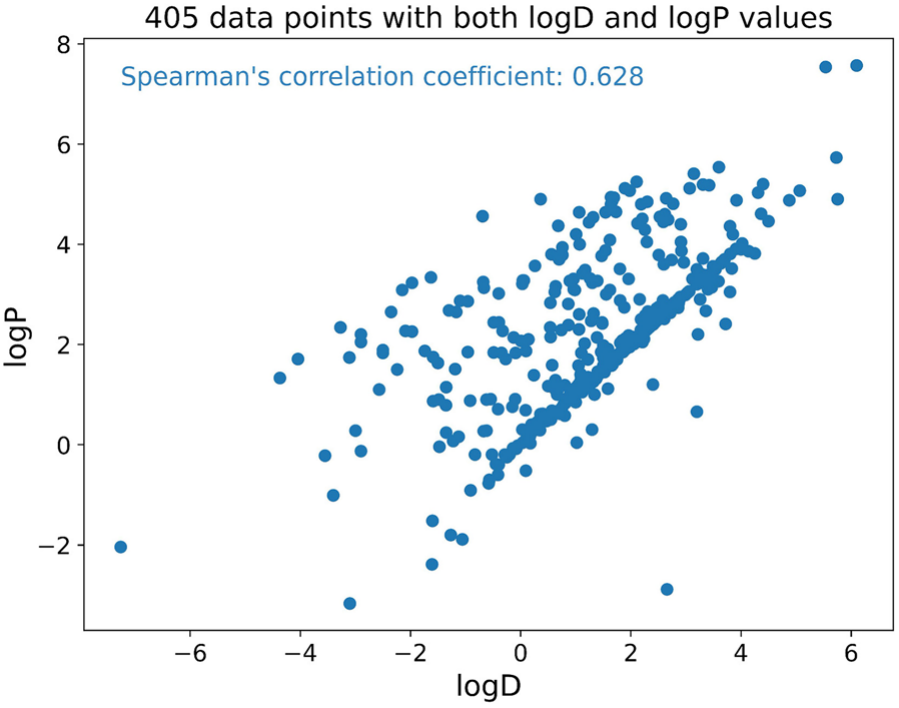
Scatter plot of experimental logP and logD values in the dataset. Spearman’s correlation coefficient values can range from − 1 to 1, where values of 1, 0, and − 1 indicate perfect positive correlation, no correlation, and perfect negative correlation, respectively

In addition to investigating the positive impact of the RT source task on logD prediction, we explored the rationale behind incorporating logP as an auxiliary task in a multitask learning approach. Multitask learning provides an inductive bias through the inclusion of auxiliary tasks, guiding the model to favor hypotheses that explain multiple tasks simultaneously. Consequently, incorporating relevant tasks can lead to improved model performance [58]. Analysis depicted in Fig. 4 demonstrates a strong positive correlation, as evidenced by a high Spearman’s correlation coefficients of 0.628 between logP and logD values for molecules. This observation suggests that integrating logP as an auxiliary task, given its monotonic relationship with logD, has the potential to enhance the accuracy of logD predictions.

In summary, the ablation studies conducted in this research highlight the importance of incorporating RT, logP, and pKa information in logD modeling. Pre-training the model with RT data allows it to be exposed to a broader chemical space, improving its ability to generalize. Furthermore, utilizing logP as a multitask provides a strong inductive bias, further improving the model’s performance. These strategies collectively contribute to the development of solutions that exhibit better generalization capabilities.

### Interpretability analysis of pKa values

**Fig. 5:**
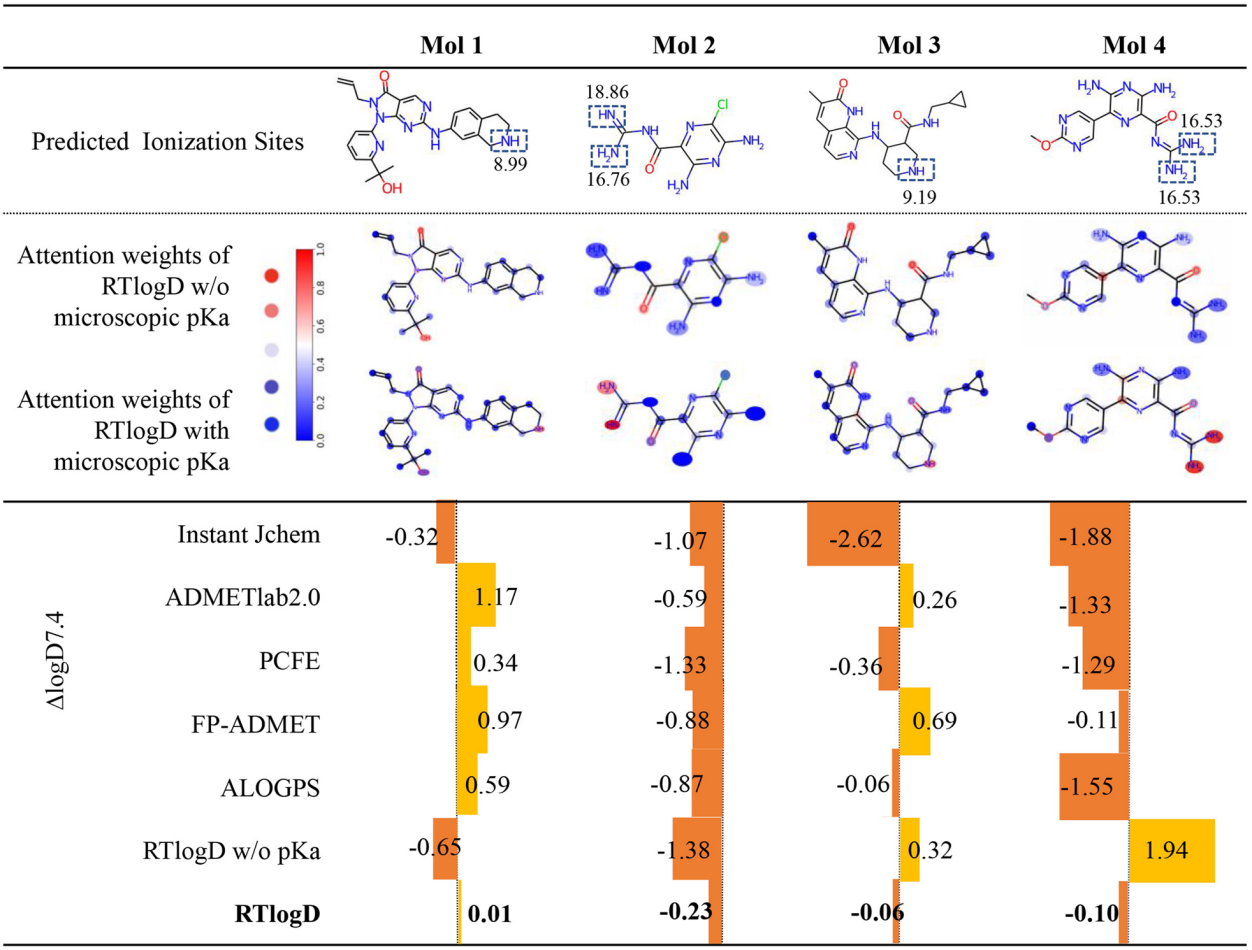
Visualization of attention weight distribution. Attention weights with blue indicates a value less than 0.5 and red indicates a value greater than 0.5 after normalization. The predicted error values of different methods are denoted by ∆logD7.4 and presented as the length of the error bars

To investigate the significance of incorporating pKa information into logD prediction, we analyzed the prediction accuracy of RTlogD and other models for highly ionizable molecules (Mol 1 to Mol 4), as depicted in Fig. 5. Predicting accurate logD values for such molecules is challenging, as ionization can alter a molecule's solubility and distribution characteristics from those of its neutral form. Highly ionizable molecules can exhibit complex pH-dependent partitioning behavior, which further complicates logD prediction.

An interpretability analysis was conducted to understand the microscopic pKa features by visualizing atomic attention. For each atom in a given molecule, attention weight scores ranging from 0 to 1 were obtained and normalized. Additionally, we created a model similar to RTlogD but without microscopic pKa as atom features (referred to as “w/o microscopic pKa” in ablation studies). Figure 5 displays the changes in atomic attention weight scores between models with and without microscopic pKa features. It is evident from Fig. 5 that the inclusion of pKa feature enables the RTlogD model to identify the strongest ionization sites in the molecules, which are assigned higher attention weights, consistent with the ionization sites (predicted by the Graph-pKa model). Consequently, RTlogD exhibits the lowest prediction errors for these challenging molecules compared to the model without pKa features and other tools. This indicates that microscopic pKa features, which reflect the ionization ability of chemical compounds and determine ionization sites, can improve logD prediction in a rational manner.

## Conclusion

In this study, we present a novel in silico logD7.4 prediction model called RTlogD. Our model combined a pre-training model on a chromatographic retention time dataset with a fine-tuning model that includes multitasks of logD and logP. We also incorporated microscopic acidic pKa and basic pKa into atomic features. Our model exhibited superior performance compared to existing tools and models, such as Instant Jchem, ADMETlab2.0, PCFE, FP-ADMET and ALOGPS. We conducted case studies and analyses to validate the strategies proposed in this paper. Our findings underscore the effectiveness of incorporating RT, logP, and microscopic pKa information, as well as utilizing transfer learning and multitask learning to enhance the performance of the RTlogD model. Pre-training the model with RT data enables it to capture a broader range of chemical space beyond the logD dataset alone. Moreover, employing RT as a multitask imparts a robust inductive bias, while incorporating microscopic pKa features provides valuable information about the compound’s ionization ability and ionization sites. These strategies contribute to the rational development of solutions that demonstrate improved generalization capabilities.

In conclusion, our study has implications for drug discovery and design, as it can make more accurate predictions of the lipophilicity of novel molecules. The reliance on high-quality internal data is crucial for achieving robust model performance. In contrast to the commercial tools employed by pharmaceutical companies, academic models often rely on literature data, which inherently carries biases and may undermine model accuracy. RTlogD aims to address the limited generalization capability of existing models caused by data scarcity. This is achieved through the implementation of pre-training and multitask learning, effectively mitigating the constraints posed by insufficient open-source data. Additionally, RTlogD employs meticulously designed descriptors that incorporate microscopic pKa features, providing essential ionization information. This incorporation contributes to enhanced generalization capabilities compared to other open-source models. In the future, we intend to periodically update RTlogD with newly available substantial datasets to ensure its adaptability. Moreover, we plan to expand our analysis to predict not only lipophilic fragments but also transformation procedures, providing alternative or improved fragment suggestions. This will be of great importance in optimizing molecular structures with moderate lipophilicity and improving the success rate of drug candidates.

### Supplementary Information

**Additional file 1:** **Table S1.** The initial atom and bond features for the RTlogD model. **Table S2.** The search ranges and optimal values of hyperparameters for the RTlogD model. **Fig S1.** Model performances on ChEMBLdb29 database. **Fig S2.** The MAE of the RTlogD model on a series of similarity subsets of T-data relative to logD training sets. **Table S3.** The search ranges and optimal values of hyperparameters for machine learning methods. **Table S4.** In-silico tools for lipophilicity prediction. **Table S5.** Ablation studies tested on logP task. **Table S6.** Comparison of Different Strategies on the logD task. **Table S7.** Performance of pre-trained RT model.

## Data Availability

All data and scripts to build the models are provided at https://github.com/WangYitian123/RTlogD.git.

## Abbreviations

RT: Retention time
RNN: Random forest
SVM: Support vector machine
ANN: Artificial neural network
XGBoost: Extreme gradient boost
MAE: Mean absolute error
RMSE: Root-mean-squared error
ECFPs: Extended connectivity fingerprints central nervous system
DL: Deep learning
ML: Machine learning
MLP: Multi-layer perceptron
QSPR: Quantitative structure–property relationship
t-SNE: T-distributed stochastic neighbor embedding

## References

[1] Waring MJ (2010). Lipophilicity in drug discovery. Expert Opin Drug Discov.

[2] Rutkowska E, Pajak K, Jozwiak K (2013). Lipophilicity–methods of determination and its role in medicinal chemistry. Acta Pol Pharm.

[3] Hughes JD, Blagg J, Price DA, Bailey S, Decrescenzo GA, Devraj RV, Ellsworth E, Fobian YM, Gibbs ME, Gilles RW (2008). Physiochemical drug properties associated with in vivo toxicological outcomes. Bioorg Med Chem Lett.

[4] Challener C (2017). Oral delivery of biologic APIs: the challenge continues. PharmTech Home.

[5] Broccatelli F, Aliagas I, Zheng H (2018). Why decreasing lipophilicity alone is often not a reliable strategy for extending IV half-life. ACS Med Chem Lett.

[6] Arnott JA, Planey SL (2012). The influence of lipophilicity in drug discovery and design. Expert Opin Drug Discov.

[7] Remko M, Boháč A, Kováčiková L (2011). Molecular structure, pKa, lipophilicity, solubility, absorption, polar surface area, and blood brain barrier penetration of some antiangiogenic agents. Struct Chem.

[8] Bhal SK, Kassam K, Peirson IG, Pearl GM (2007). The rule of five revisited: applying log D in place of log P in drug-likeness filters. Mol Pharm.

[9] Yang ZY, Yang ZJ, Dong J, Wang LL, Zhang LX, Ding JJ, Ding XQ, Lu AP, Hou TJ, Cao DS (2019). Structural analysis and identification of colloidal aggregators in drug discovery. J Chem Inf Model.

[10] Andrés A, Rosés M, Ràfols C, Bosch E, Espinosa S, Segarra V, Huerta JM (2015). Setup and validation of shake-flask procedures for the determination of partition coefficients (log D) from low drug amounts. Eur J Pharm Sci.

[11] Donovan SF, Pescatore MC (2002). Method for measuring the logarithm of the octanol–water partition coefficient by using short octadecyl–poly (vinyl alcohol) high-performance liquid chromatography columns. J Chromatogr A.

[12] Ràfols C, Subirats X, Rubio J, Rosés M, Bosch E (2017). Lipophilicity of amphoteric and zwitterionic compounds: a comparative study of determination methods. Talanta.

[13] Venkatraman V (2021). FP-ADMET: a compendium of fingerprint-based ADMET prediction models. J Cheminform.

[14] Xiong G, Wu Z, Yi J, Fu L, Yang Z, Hsieh C, Yin M, Zeng X, Wu C, Lu A (2021). ADMETlab 2.0: an integrated online platform for accurate and comprehensive predictions of ADMET properties. Nucleic Acids Res.

[15] Lapins M, Arvidsson S, Lampa S, Berg A, Schaal W, Alvarsson J, Spjuth O (2018). A confidence predictor for logD using conformal regression and a support-vector machine. J Cheminform.

[16] Galushka M, Swain C, Browne F, Mulvenna MD, Bond R, Gray D (2021). Prediction of chemical compounds properties using a deep learning model. Neural Comput Appl.

[17] Fu L, Liu L, Yang ZJ, Li P, Ding JJ, Yun YH, Lu AP, Hou TJ, Cao DS (2020). Systematic modeling of log D(7.4) based on ensemble machine learning, group contribution, and matched molecular pair analysis. J Chem Inf Model.

[18] Xiong Z, Wang D, Liu X, Zhong F, Wan X, Li X, Li Z, Luo X, Chen K, Jiang H (2020). Pushing the boundaries of molecular representation for drug discovery with the graph attention mechanism. J Med Chem.

[19] Tang B, Kramer ST, Fang M, Qiu Y, Wu Z, Xu D (2020). A self-attention based message passing neural network for predicting molecular lipophilicity and aqueous solubility. J Cheminform.

[20] Hasebe T (2021). Knowledge-embedded message-passing neural networks: improving molecular property prediction with human knowledge. ACS Omega.

[21] Su Y, Shen W, Ren J, Shen W, Man Y, Dong L (2021). Deep learning in QSPR modeling for the prediction of critical properties. Applications of artificial intelligence in process systems engineering.

[22] Su Y, Wang Z, Jin S, Shen W, Ren J, Eden MR (2019). An architecture of deep learning in QSPR modeling for the prediction of critical properties using molecular signatures. AlChE J.

[23] Göller AH, Kuhnke L, Montanari F, Bonin A, Schneckener S, Ter Laak A, Wichard J, Lobell M, Hillisch A (2020). Bayer’s in silico ADMET platform: a journey of machine learning over the past two decades. Drug Discov Today.

[24] Wolkenhauer O (2020). Systems medicine: integrative, qualitative and computational approaches.

[25] Feinberg EN, Joshi E, Pande VS, Cheng AC (2020). Improvement in ADMET prediction with multitask deep featurization. J Med Chem.

[26] Wu J, Wang J, Wu Z, Zhang S, Deng Y, Kang Y, Cao D, Hsieh CY, Hou T (2022). ALipSol: an attention-driven mixture-of-experts model for lipophilicity and solubility prediction. J Chem Inf Model.

[27] Bergazin TD, Tielker N, Zhang Y, Mao J, Gunner MR, Francisco K, Ballatore C, Kast SM, Mobley DL (2021). Evaluation of log P, pK(a), and log D predictions from the SAMPL7 blind challenge. J Comput Aided Mol Des.

[28] Livingstone DJ (2003). Theoretical property predictions. Curr Top Med Chem.

[29] Pan SJ, Yang Q (2010). A survey on transfer learning. IEEE Trans Knowl Data Eng.

[30] Caruana R (1997). Multitask learning. Mach Learn.

[31] Aliagas I, Gobbi A, Lee ML, Sellers BD (2022). Comparison of logP and logD correction models trained with public and proprietary data sets. J Comput Aided Mol Des.

[32] Lukashina N, Alenicheva A, Vlasova E, Kondiukov A, Khakimova A, Magerramov E, Churikov N, Shpilman A (2020). Lipophilicity prediction with multitask learning and molecular substructures representation. arXiv.

[33] Wieder O, Kuenemann M, Wieder M, Seidel T, Meyer C, Bryant SD, Langer T (2021). Improved lipophilicity and aqueous solubility prediction with composite graph neural networks. Molecules.

[34] Parinet J (2021). Predicting reversed-phase liquid chromatographic retention times of pesticides by deep neural networks. Heliyon.

[35] Win ZM, Cheong AMY, Hopkins WS (2023). Using machine learning to predict partition coefficient (Log P) and distribution coefficient (Log D) with molecular descriptors and liquid chromatography retention time. J Chem Inf Model.

[36] Domingo-Almenara X, Guijas C, Billings E, Montenegro-Burke JR, Uritboonthai W, Aisporna AE, Chen E, Benton HP, Siuzdak G (2019). The METLIN small molecule dataset for machine learning-based retention time prediction. Nat Commun.

[37] Duan Y-J, Fu L, Zhang X-C, Long T-Z, He Y-H, Liu Z-Q, Lu A-P, Deng Y-F, Hsieh C-Y, Hou T-J (2023). Improved GNNs for Log D7.4 prediction by transferring knowledge from low-fidelity data. J Chem Inf Model.

[38] Tetko IV, Tanchuk VY (2002). Application of associative neural networks for prediction of lipophilicity in ALOGPS 2.1 program. J Chem Inf Comput Sci.

[39] ChemAxon Marvin Suite;. ChemAxon Inc, 2017.

[40] Mendez D, Gaulton A, Bento AP, Chambers J, De Veij M, Félix E, Magariños MP, Mosquera JF, Mutowo P, Nowotka M (2019). ChEMBL: towards direct deposition of bioassay data. Nucleic Acids Res.

[41] Landrum G (2013). Rdkit documentation. Release.

[42] Wu Z, Ramsundar B, Feinberg EN, Gomes J, Geniesse C, Pappu AS, Leswing K, Pande V (2018). MoleculeNet: a benchmark for molecular machine learning. Chem Sci.

[43] The Physical Properties Database (PHYSPROP) by Syracuse Research Corporation (SRC) https://www.srcinc.com/what-we-do/environmental/scientific-databases.html

[44] Ihlenfeldt WD, Voigt JH, Bienfait B, Oellien F, Nicklaus MC (2002). Enhanced CACTVS browser of the open NCI database. J Chem Inf Comput Sci.

[45] Sushko I, Novotarskyi S, Korner R, Pandey AK, Rupp M, Teetz W, Brandmaier S, Abdelaziz A, Prokopenko VV, Tanchuk VY (2011). Online chemical modeling environment (OCHEM): web platform for data storage, model development and publishing of chemical information. J Comput Aided Mol Des.

[46] Martel S, Gillerat F, Carosati E, Maiarelli D, Tetko IV, Mannhold R, Carrupt PA (2013). Large, chemically diverse dataset of logP measurements for benchmarking studies. Eur J Pharm Sci.

[47] Rigatti SJ (2017). Random forest. J Insur Med.

[48] Noble WS (2006). What is a support vector machine?. Nat Biotechnol.

[49] Jain AK, Mao J, Mohiuddin KM (1996). Artificial neural networks: a tutorial. Computer.

[50] Chen T, He T, Benesty M, Khotilovich V, Tang Y, Cho H, Chen K, Mitchell R, Cano I, Zhou T (2015). Xgboost: extreme gradient boosting. R Package Version.

[51] Pedregosa F, Varoquaux G, Gramfort A, Michel V, Thirion B, Grisel O, Blondel M, Prettenhofer P, Weiss R, Dubourg V (2011). Scikit-learn: machine learning in python. J Mach Learn Res.

[52] Li M, Zhou J, Hu J, Fan W, Zhang Y, Gu Y, Karypis G (2021). Dgl-lifesci: an open-source toolkit for deep learning on graphs in life science. ACS Omega.

[53] Xiong J, Li Z, Wang G, Fu Z, Zhong F, Xu T, Liu X, Huang Z, Liu X, Chen K (2022). Multi-instance learning of graph neural networks for aqueous pKa prediction. Bioinformatics.

[54] Yang Q, Ji H, Lu H, Zhang Z (2021). Prediction of liquid chromatographic retention time with graph neural networks to assist in small molecule identification. Anal Chem.

[55] Ramsundar B, Liu B, Wu Z, Verras A, Tudor M, Sheridan RP, Pande V (2017). Is Multitask deep learning practical for pharma?. J Chem Inf Model.

[56] Rogers D, Hahn M (2010). Extended-connectivity fingerprints. J Chem Inf Model.

[57] Lvd M, Hinton GE (2008). Visualizing data using t-SNE. J Mach Learn Res.

[58] Ruder S (2017). An overview of multi-task learning in deep neural networks. arXiv.

