# Supplementary material for: LogD7.4 prediction enhanced by transferring knowledge from chromatographic retention time, microscopic pKa and logP
Source: J Cheminform. 2023 Sep 5;15:76. doi: 10.1186/s13321-023-00754-4 (PMC10478446; doi:10.1186/s13321-023-00754-4)
Supplement: Supplementary file 1 — Additional file 1: Table S1. The initial atom and bond features for the RTlogD model. Table S2. The search ranges and optimal values of hyperparameters for the RTlogD model. Fig S1. Model performances on ChEMBLdb29 database. Fig S2. The MAE of the RTlogD model on a series of similarity subsets of T-data relative to logD training sets. Table S3. The search ranges and optimal values of hyperparameters for machine learning methods. Table S4. In-silico tools for lipophilicity prediction. Table S5. Ablation studies tested on logP task. Table S6. Comparison of Different Strategies on the logD task. Table S7. Performance of pre-trained RT model. [file 13321_2023_754_MOESM1_ESM.docx]

Additional file

LogD7.4 Prediction Enhanced by Transferring Knowledge from Chromatographic Retention Time, Microscopic pKa and logP

*Yitian Wang^1,2^**,* *Jiacheng Xiong^1,2^, Fu Xiao^3,1^, Wei Zhang^1,2^, Kaiyang Cheng^3,1^**, Jingxin Rao^1,2^, Buying Niu^1,2^, Xiaochu Tong^1,2^, Ning Qu^1,2^, Runze Zhang^1,2^, Dingyan Wang^4^, Kaixian Chen^1,2,3^, Xutong Li^1,2,*^_，_and Mingyue Zheng^1,2,3,*^*

*Correspondence: [myzheng@simm.ac.cn](mailto:myzheng@simm.ac.cn); [lixutong@simm.ac.cn](mailto:lixutong@simm.ac.cn)

^1^ Drug Discovery and Design Center, State Key Laboratory of Drug Research, Shanghai Institute of Materia Medica, Chinese Academy of Sciences, 555 Zuchongzhi Road, Shanghai 201203, China

^2^ University of Chinese Academy of Sciences, No. 19A Yuquan Road, Beijing 100049, China

^3^ Nanjing University of Chinese Medicine, 138 Xianlin Road, Nanjing 210023, China

^4^ Lingang Laboratory, Shanghai 200031, China

**Table of Contents**

| **Table S1** The initial atom and bond features for the RTlogD model. | S3 |
| --- | --- |
| **Table S2** The search ranges and optimal values of hyperparameters for the RTlogD model. | S4 |
| **Fig. S1** Model performances on ChEMBLdb29 database. | S4 |
| **Fig. S2** The MAE of the RTlogD model on a series of similarity subsets of T-data relative to logD training sets. | S5 |
| **Table S3** The search ranges and optimal values of hyperparameters for machine learning methods. | S5 |
| **Table S4** In-silico tools for lipophilicity prediction. | S6 |
| **Table S5** Ablation studies tested on logP task. | S6 |
| **Table S6** Comparison of Different Strategies on the logD task. | S7 |
| **Table S7** Performance of pre-trained RT model. | S8 |
| **References** | S8 |

**Table** **S1** The initial atom and bond features for the RTlogD model.

| **Atom feature** | **Size** | **Description** |
| --- | --- | --- |
| atom symbol | 43 | [C, N, O, S, F, Si, P, Cl, Br, Mg, Na, Ca, Fe, As, Al, I, B, V, K, Tl, Yb, Sb, Sn, Ag, Pd, Co, Se, Ti, Zn, H, Li, Ge, Cu, Au, Ni, Cd, In, Mn, Zr, Cr, Pt, Hg, Pb] (one-hot) |
| degree | 11 | number of covalent bonds (0-10, one-hot) |
| implicit hydrogens | 7 | number of implicit hydrogens (0-6 one-hot) |
| formal charge | 1 | electrical charge (integer) |
| radical electrons | 1 | number of radical electrons (integer) |
| hybridization | 5 | [sp, sp2, sp3, sp3d, sp3d2] (one-hot) |
| aromaticity | 1 | whether the atom is aromatic (one-hot) |
| hydrogens | 5 | number of connected hydrogens (0-4, one-hot) |
| microscopic pKa | 2 | acidic pKa and basic pKa(0-1,real number) |
| **Bond feature** | **Size** | **Description** |
| bond type | 4 | [single, double, triple, aromatic] (one-hot) |
| conjugation | 1 | whether the bond is conjugated |
| ring | 1 | whether the bond is in ring |
| stereo | 6 | [StereoNone, StereoAny, StereoZ, StereoE, StereoCIS, StereoANS] (one-hot) |

**Table S2** The search ranges and optimal values of hyperparameters for the RTlogD model.

| **type** | **Hyperparameter** | **Possible values** | **optimal value** |
| --- | --- | --- | --- |
| With RT pre-trained | Layer_num | 3,4,5,6,7 | 3 |
|  | num_timesteps | 1,2,3 | 1 |
|  | graph_feat_size | 100,200,300,400 | 300 |
|  | weight_decay | 0.0003,0.003 | 0.0003 |
|  | learning_rate | 0.001, 0.01,0.0001 | 0.001 |
|  | dropout | 0, 0. 1, 0.2, 0.3, 0.4, 0.5 | 0.2 |
| Without pre-trained | Layer_num | 3,4,5,6,7 | 3 |
|  | num_timesteps | 1,2,3 | 1 |
|  | graph_feat_size | 100,200,300,400 | 300 |
|  | weight_decay | 0.0003,0.003 | 0.0003 |
|  | learning_rate | 0.001, 0.01,0.0001 | 0.0001 |
|  | dropout | 0, 0. 1, 0.2, 0.3, 0.4, 0.5 | 0.2 |


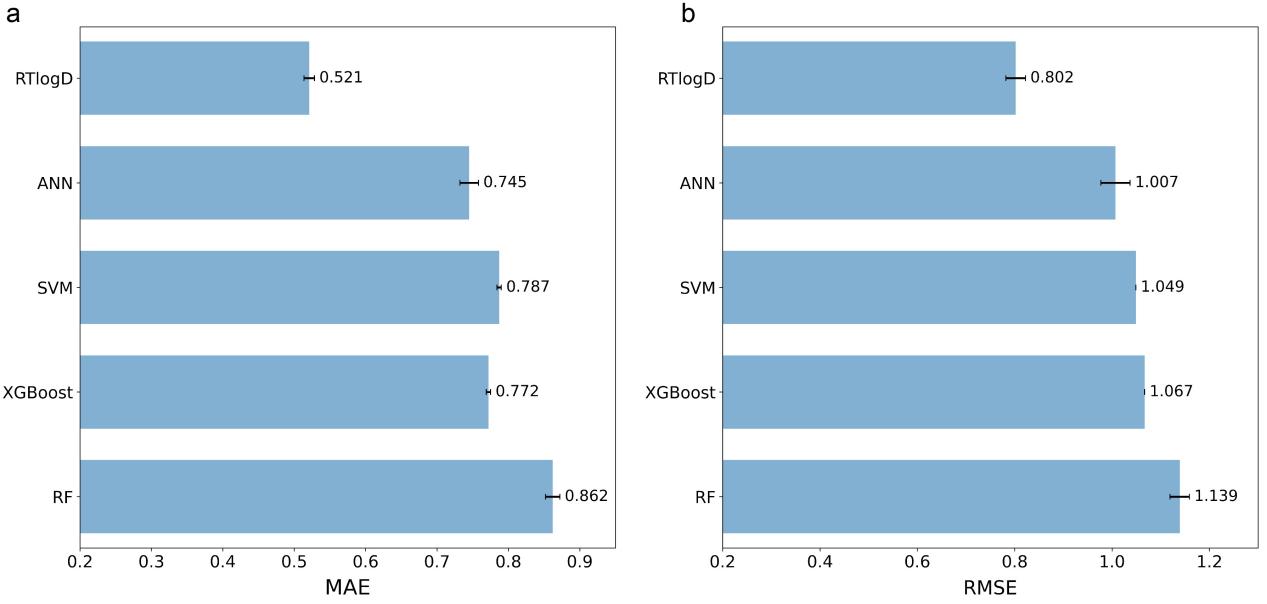


**Fig. S1** Model performances on ChEMBLdb29 database. a The MAE of those models on the ChEMBLdb29 database. b The RMSE of those models on the ChEMBLdb29 database.

This database was divided into training, validation, and test sets using the scaffold split method. To evaluate the performance of RTlogD, four conventional machine learning models and CALlogD were implemented and taken as benchmark methods. In terms of machine learning methods, both ANN and XGBoost achieved relatively favourable results. Remarkably, the RTlogD model outperformed the traditional machine learning models, indicating that the deep learning approach was superior for this logD prediction task.


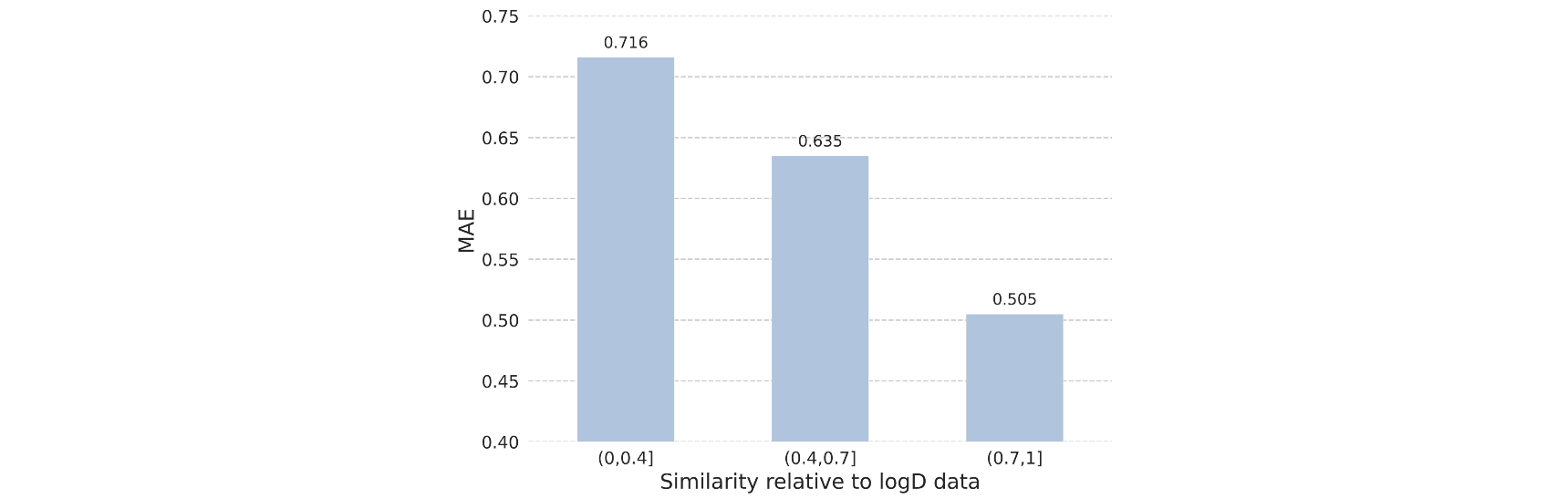


**Fig. S2** The MAE of the RTlogD model on a series of similarity subsets of T-data relative to logD training sets.

**Table S3** The search ranges and optimal values of hyperparameters for machine learning methods.

| **model** | **Hyperparameter** | **Possible values** | **optimal value** |
| --- | --- | --- | --- |
| SVM | kernel | ‘linear’, ‘rdf’ | 'rdf' |
|  | gamma | 0.00001, 0.0001, 0.001, 0.01,0.1,1,10 | 0.001 |
|  | C | 0.1,1,10,100,1000,10000,100000 | 100 |
| RF | n_estimators | 30,50,70,100,150,300 | 300 |
|  | max_depth | 3,5,10,15,20,30, None | None |
|  | min_samples_split | 2,3,4,5 | 2 |
| ANN | Layer_size | 50,100,200,400,800 | 800 |
|  | Layer_num | 2,3,4 | 4 |
|  | alpha | 0.00003,0.0001,0.0003,0.001,0.003 | 0.003 |
|  | learning_rate_init | 0.0001, 0.001, 0.01 | 0.001 |
| XGBoost | n_estimators | 50,100,300,500,1000,2000,3000 | 2000 |
|  | max_depth | 3,4,6,9 | 3 |
|  | subsample | 1.0,0.9,0.8,0.5 | 0.9 |

**Table S4** In-silico tools for lipophilicity prediction.

| **Tools** | **Methods** | **logD** | **logP** | **URL** |
| --- | --- | --- | --- | --- |
| Instant Jchem [1] | Atom contribution | √ | √ | <https://chemaxon.com/calculators-and-> predictors |
| ADMETlab 2.0 [2] | Graph-based methods | √ | √ | https://admetmesh.scbdd.com/ |
| PCFE [3] | Graph-based methods | √ |  | https:// gitlab.com/vishsoft/fpadmet |
| FP-ADMET [4] | Random forest | √ | √ | https:// gitlab.com/vishsoft/fpadmet |
| ALOGPS [5] | Associative  neural networks | √ | √ | http://www.vcclab.org/lab/alogps |

**Table S5** Ablation studies tested on logP task.

| Method | **RMSE** | **MAE** | **R2** |
| --- | --- | --- | --- |
| baseline | 0.450 | 0.342 | 0.742 |
| w/o pre-training | 0.517 | 0.448 | 0.659 |
| w/o microscopic pKa | 0.456 | 0.314 | 0.735 |
| w/o pre-training and microscopic pKa | 0.570 | 0.413 | 0.586 |

The analysis of the results demonstrates that the model performance for the logP prediction task remains unchanged even in the absence of microscopic pKa features, suggesting that there is no correlation between ionization and the logP task. It is due to the fact that logP measures the lipophilicity of molecules in neutral form. By contrast, a significant degradation in the model performance was observed when the CRT pretraining was excluded, indicating that chromatographic retention time is also associated with the logP task, as demonstrated by the performance on the training set.

**Table S6** Comparison of Different Strategies on the logD task.

| **strategies** | **RMSE** | **MAE** | **R2** |
| --- | --- | --- | --- |
| baseline | **0.923** | **0.694** | **0.550** |
| macroscopic pKa | 0.980 | 0.722 | 0.492 |
| swap strategies of pretraining and multitask | 1.034 | 0.731 | 0.435 |
| all the data modeled in multitask way | 1.080 | 0.748 | 0.384 |

We modified our models in the following three ways separately, maintaining all other condition unchanged for logD task prediction. Firstly, in order to determine whether microscopic pKa is more suitable than macroscopic pKa, we carried out experiments that utilized both basic and acidic pKa of the molecules in multitask way parallel with logP and logD tasks. Secondly, we attempted to swap strategies of pretraining and multitask. That is to say, we tried to employ logP pretraining and chromatographic retention time is used in multitask way. Thirdly, we constructed the model using all the data by the application of multitask learning without pretraining.

It can be found that the implementation of all three strategies will result in a decrease in the performance of the model. In terms of macroscopic pKa, we assumed that it may be not as specific as microscopic pKa and the information which provided is not abundant. Therefore, the performance of the model declined. Likewise, the model doesn't work as well as before when we swapped strategies of pretraining and multitask. The availability of logP data is not as extensive as that of chromatography data, making chromatography data a more appropriate choice for pretraining purposes. Employing all the data for multi-task modeling does not benefit the model performance as there is a significant difference in the amount of different modelling tasks, especially when there is a large amount of chromatography data.

**Table S7** Performance of pre-trained RT model.

| **Methods** | **MAE** | **R2** |
| --- | --- | --- |
| GNN-RT | 39.87 | 0.85 |
| RT | 40.39 | 0.86 |

**References**

1. ChemAxon Marvin Suite;. ChemAxon Inc, 2017.

2. Xiong G, Wu Z, Yi J, Fu L, Yang Z, Hsieh C, Yin M, Zeng X, Wu C, Lu A et al (2021) ADMETlab 2.0: an integrated online platform for accurate and comprehensive predictions of ADMET properties. Nucleic Acids Res 49:W5-W14.

3. Duan YJ, Fu L, Zhang XC, Long TZ, He YH, Liu ZQ, Lu AP, Deng YF, Hsieh CY, Hou TJ et al (2023) Improved GNNs for Log D(7.4) Prediction by Transferring Knowledge from Low-Fidelity Data. J Chem Inf Model 63:2345–2359.

4. Venkatraman V (2021) FP-ADMET: a compendium of fingerprint-based ADMET prediction models. J Cheminform 13:75.

5. Tetko IV, Tanchuk VY (2002) Application of associative neural networks for prediction of lipophilicity in ALOGPS 2.1 program. J Chem Inf Comput Sci 42:1136-1145.
